# Supplementary material for: Antagonistic Roles of the Tumor Suppressor miR-210-3p and Oncomucin MUC4 Forming a Negative Feedback Loop in Pancreatic Adenocarcinoma
Source: Cancers (Basel). 2021 Dec 9;13(24):6197. doi: 10.3390/cancers13246197 (PMC8699468; doi:10.3390/cancers13246197)
Supplement: Supplementary file 1 [file cancers-13-06197-s001.zip › cancers-1457794-supplementary.pdf]

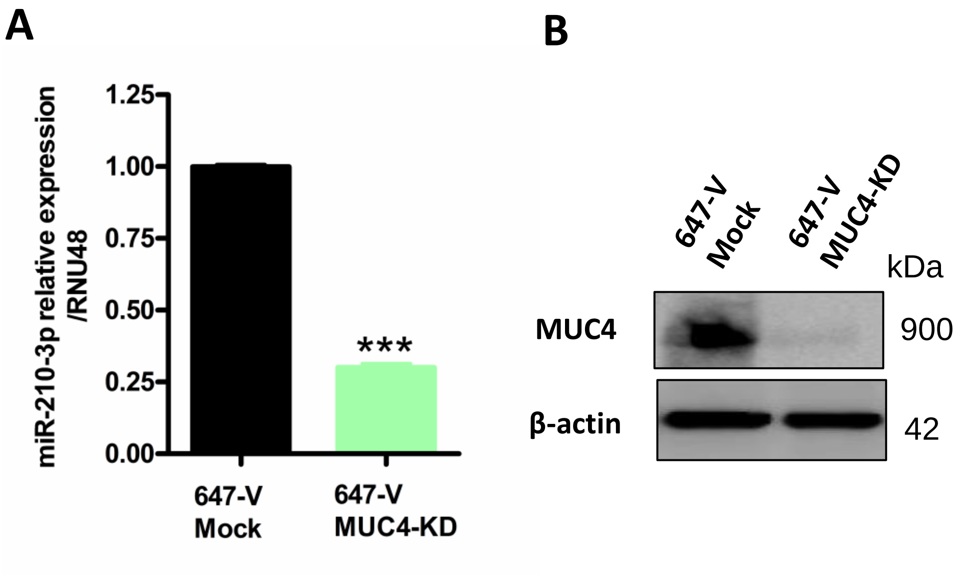

Figure S1: MUC4 inhibition induced a decrease of miR-210-3p relative level in 647-V cells. (A) qRT–PCR analysis of miR-210-3p relative expression was performed in mock and MUC4-KD 647-V cells. Expression of miR-210 was evaluated according to the $2^{-\Delta Ct}$ method ($\Delta\Delta$Ct= (Ct miR-210 - Ct RNU48). (**B**) Western blotting analysis of MUC4 and β-actin expression in 647-V mock and MUC4-KD cells.

A

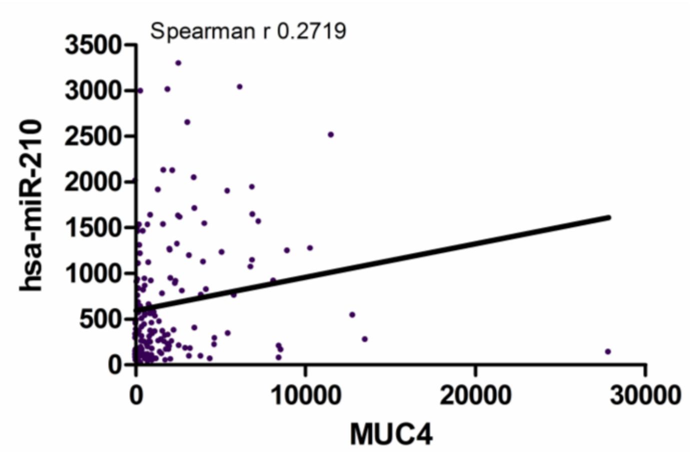

Figure S2: MUC4 and miR-210 expression levels are correlated in TCGA-PAAD dataset. MiR-210 (RPM) and MUC4 RNA (RSEM) relative expression levels were extracted using www.linkedomics.org, where after Spearman correlation test were performed.

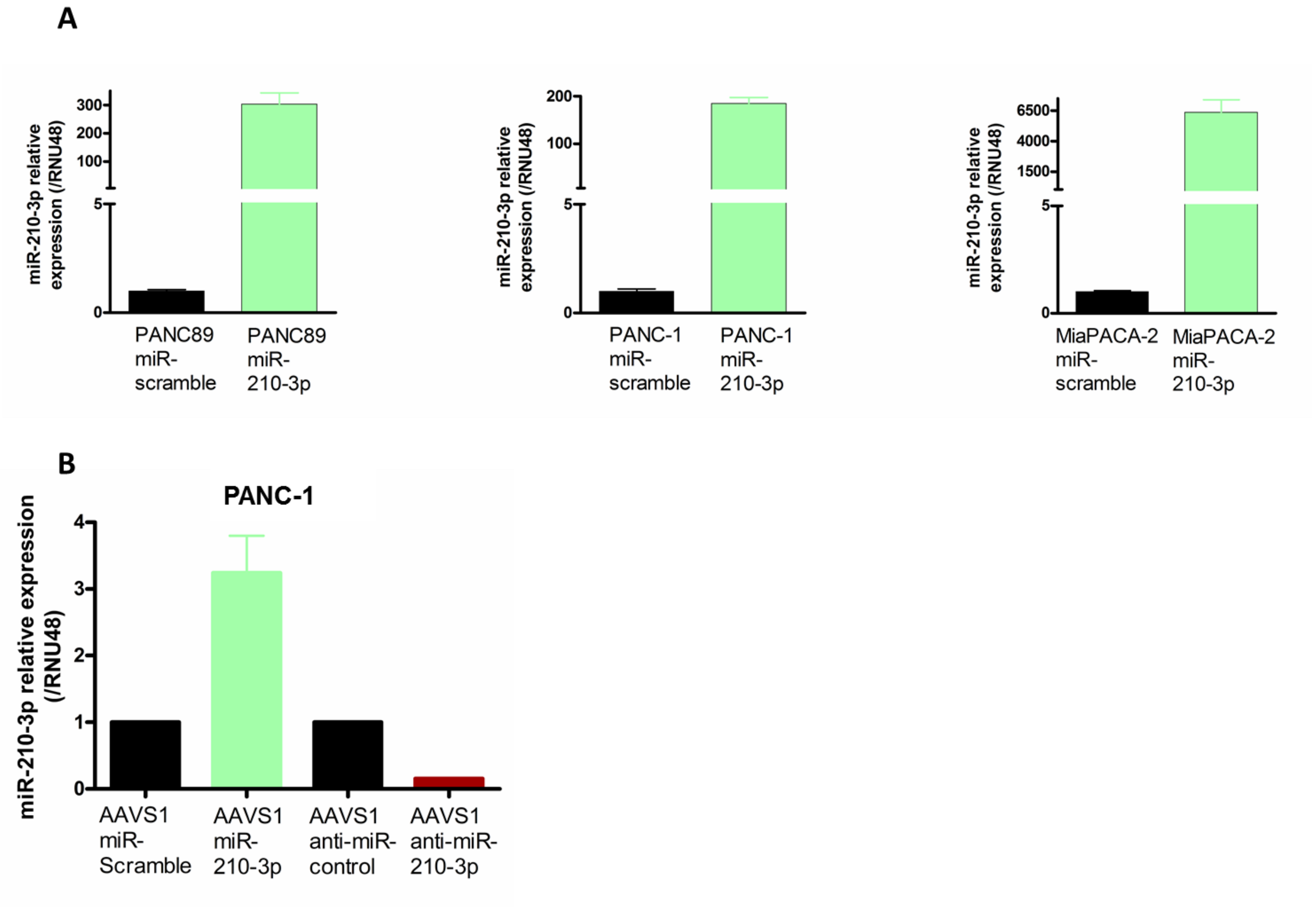

Figure S3: Evaluation of miR-210 expression in transient and stable expression cell models. qRT–PCR analysis of miR-210-3p relative expression in PANC89, PANC1 and MIA PaCa-2 pancreatic cancer cells 96 h post miR-210 transfection (**A**) and in PANC1 stably transfected with miR-210-3p, anti-miR-210-3p and miR-scramble, anti-miR-control (PANC-1/AAVS1) (**B**).

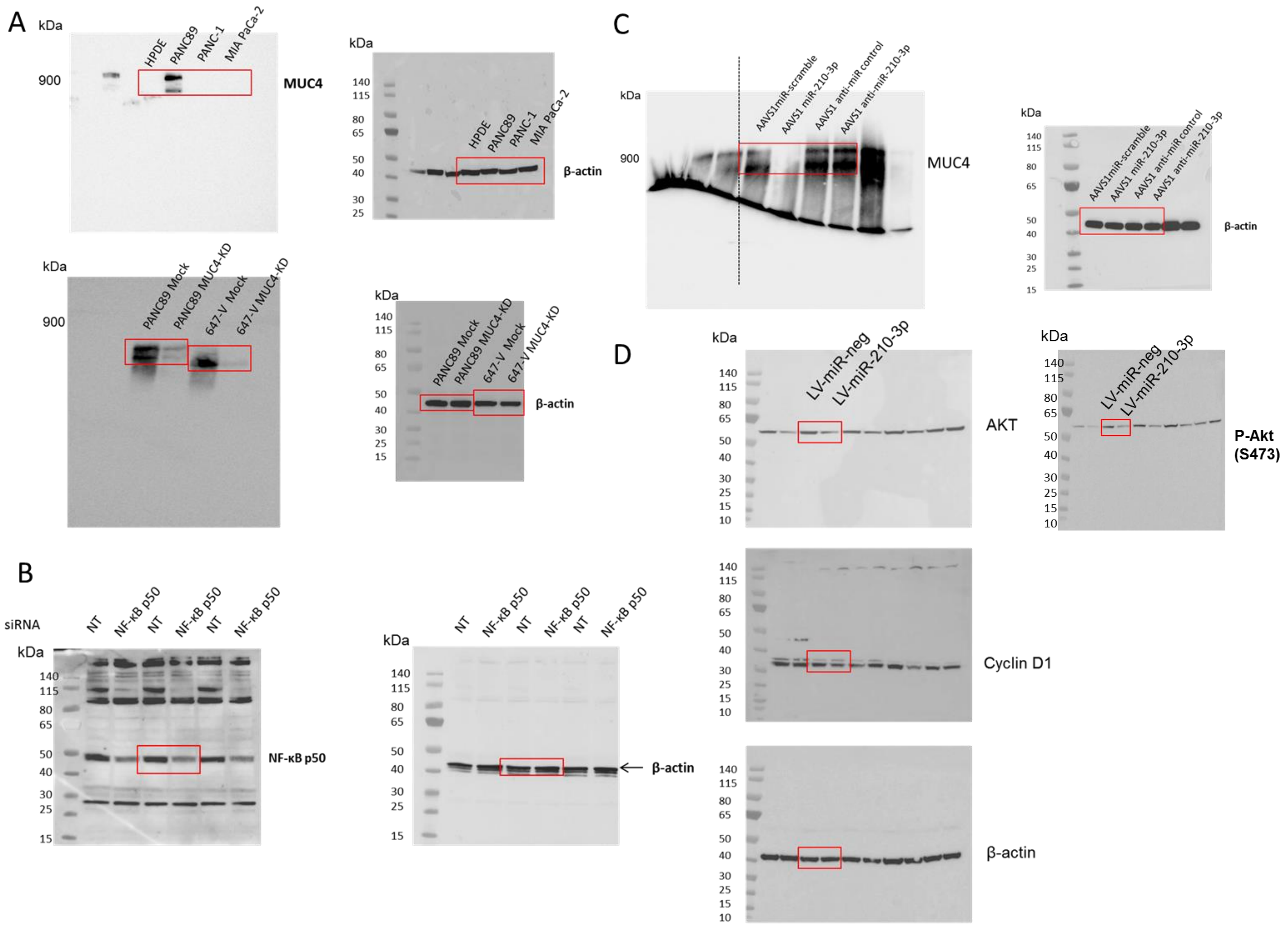

Figure S4: Full pictures of Western blots. (A) MUC4 and β-actin uncropped western blots from figure 1 and S1. (B) NF-κB p 50 and β-actin uncropped western blots from figure 3. (C) MUC4 and β-actin uncropped western blots from figure 4. (D) Akt, cyclinD1 and β-actin uncropped western blots from figure 7.
